# Spatial Metabolomics and Single-Cell Virtual Knockout Screening Reveal Solanesol Improves Parkinson’s Disease-like Pathology Based on Lipid Inflammation Mechanism

**DOI:** 10.3390/metabo16080541

**Published:** 2026-07-31

**Authors:** Qian Li, Lutao Xu, Mingyu Zhu, Gaoge Wang, Huan Chen, Hongwei Hou, Yu Bai

**Affiliations:** 1 Beijing National Laboratory for Molecular Sciences, Key Laboratory of Bioorganic Chemistry and Molecular Engineering of Ministry of Education, College of Chemistry and Molecular Engineering, Peking University, Beijing 100871, China; noone639@163.com; 2 Beijing Life Science Academy, Beijing 102209, China; xuebixuebia@163.com (L.X.); zmy06052021@163.com (M.Z.); wanggg@blsa.com.cn (G.W.); 3 Key Laboratory of Tobacco Biological Effects, China National Tobacco Quality Supervision & Test Center, Zhengzhou 450099, China

**Keywords:** spatial metabolomics, single-cell virtual knockout, Mendelian randomization, Solanesol, Parkinson’s disease, neuroinflammation

## Abstract

**Background**: Parkinson’s disease (PD) is characterized by a complex interplay of dopaminergic degeneration, glial activation, and lipid metabolic dysregulation. However, accurately describing how natural product interventions remodel these pathologies across distinct brain regions and cellular microenvironments remains a critical challenge. **Methods**: We established an integrated multi-omics framework to decode the neuroprotective mechanisms of solanesol (Sol) in an MPTP-induced PD mouse model. We combined single-cell eQTL-based Mendelian randomization (scMR), transcriptomic localization, and virtual knockout analyses to prioritize cell-type-specific regulatory nodes across neuronal, glial, and vascular populations, avoiding the limitations of traditional bulk targeting. In vivo behavioral assays were conducted, alongside orthogonal validation via airflow-assisted desorption electrospray ionization mass spectrometry imaging (AFADESI-MSI) and gene–metabolite co-enrichment analysis, to map regional metabolic networks and structural spatial reprogramming. **Results**: Computational prioritization highlighted cell-type-specific regulatory nodes including PRKCB, PRKCE, PDGFRB, and FABP3/5. In vivo, Sol attenuated motor and cognitive deficits and largely restored the highly compartmentalized spatial distributions of striatal dopamine, L-DOPA, and acetylcholine. Crucially, AFADESI-MSI and co-enrichment analysis revealed that Sol specifically reversed MPTP-induced spatial disruptions by rescuing key neuromodulatory metabolites—including cervonoyl ethanolamide, phosphatidylcholine species, taurine, and NADHX—which were tightly coupled to sphingolipid signaling, fatty-acid transport, mitochondrial translation, and cell-adhesion pathways. Conclusions: Sol ameliorates PD-like pathology not through a singular target, but by choreographing a spatially and cellularly compartmentalized restoration of lipid–inflammatory homeostasis. Furthermore, our integrated single-cell and spatial metabolomic blueprint sets a new methodological paradigm for elucidating the precise execution programs of natural neurotherapeutics.

## 1. Introduction

Parkinson’s disease (PD) is one of the most common neurodegenerative movement disorders, characterized by progressive loss of dopaminergic neurons in the substantia nigra pars compacta, reduced striatal dopaminergic input, as well as motor manifestations such as bradykinesia, rigidity, resting tremor, and postural instability. With population aging, the global burden of PD continues to increase, but current therapeutic strategies are still limited to symptomatic treatment [1,2,3,4]. L-DOPA can improve motor symptoms but is frequently associated with long-term exercise complications and cannot prevent neurodegeneration. Mechanistically, PD involves convergent changes in neuronal vulnerability, glial activation, mitochondrial dysfunction, redox imbalance, proteostasis disruption, and lipid metabolic remodeling [5,6]. Membrane phospholipids, sphingolipids, fatty acid derivatives, and choline metabolites are increasingly recognized as critical coordinators of synaptic function, inflammatory signaling, and specific cell-death pathways, predominantly driving lipid-peroxidation-mediated ferroptosis, ceramide-dependent apoptosis, and necroptotic membrane disruption [7,8,9,10,11]. The inherent limitations of current symptomatic treatments fundamentally fail to arrest underlying neurodegeneration, highlighting the urgency of discovering novel, disease-modifying therapeutics. The mammalian brain operates through highly compartmentalized and functionally diverse microenvironments; bulk tissue analyses often obscure the precise loci of pharmacological action. Consequently, a major unresolved challenge remains delineating exactly how candidate interventions reshape disease-related molecular abnormalities within their native cellular and spatial contexts.

Solanesol (Sol), an all-trans-polyprenol consisting of nine isoprene units (C45), serves as the essential lipophilic side-chain precursor for the semi-synthesis of macrocyclic quinone cofactors, such as coenzyme Q10 and vitamin K2. Mechanistically, because Coenzyme Q10 (ubiquinone-10) requires a decaprenyl (C50) chain, solanesol typically undergoes a one-isoprene unit (C5) elongation phase—such as bromination followed by coupling with a C5 synthon—or is directly coupled with a C5-extended 2,3-dimethoxy-5-methyl-1,4-benzoquinone derivative via Lewis-acid-catalyzed alkylation [12]. Conversely, for Vitamin K2, solanesol’s C45 skeleton matches the exact structural requirement for the nonaprenyl side chain of menaquinone-9 (MK-9). This industrial synthesis proceeds through the Friedel-Crafts alkylation of a protected menadiol core with activated solanesol, followed by selective ceric ammonium nitrate (CAN) or silver oxide oxidation to reconstruct the active bio-quinone architecture [13]. Sol may therefore be relevant to lipid–inflammatory regulation and neuroprotection [14]. However, database-based target prediction often yields a multitude of broad candidate nodes, while conventional bulk omics inevitably averages out crucial localized signals across anatomically and metabolically heterogeneous brain regions. The tissue dilution effect obscures the precise relationships among Sol’s targets, cell-type contexts, spatial metabolic footprints, and lipid–inflammatory networks in PD-like pathology. Consequently, determining the functional relevance of candidate targets and pinpointing the exact anatomical loci of metabolic recovery remains a formidable challenge. To address the loss of spatial architecture, mass spectrometry imaging (MSI), the cornerstone of spatial metabolomics, has emerged as a transformative analytical tool. Cutting-edge MSI modalities, such as matrix-assisted laser desorption/ionization (MALDI) [15,16], desorption electrospray ionization (DESI) [17,18], and airflow-assisted desorption electrospray ionization (AFADESI) [19,20], enable the in situ visualization of neurotransmitters, lipids, and small molecules directly on intact brain sections, seamlessly linking molecular abundance with precise anatomical localization. In parallel, single-cell sequencing has revolutionized our capacity to decode complex tissue heterogeneity, allowing researchers to precisely assign disease-associated molecular signatures to specific neuronal and glial populations [21]. To transition from static transcriptomic mapping to dynamic functional prioritization, single-cell virtual knockout screening has recently been developed as an advanced computational strategy [22]. By computationally deleting specific candidate genes within inferred single-cell gene regulatory networks, this technique predicts downstream functional perturbations, enabling the rapid identification of cell-type-specific therapeutic nodes without the immediate need for exhaustive in vivo genetic engineering. When spatial metabolomics is strategically integrated with single-cell expression localization and single-cell virtual knockout screening, it becomes possible to ask not only which genes or metabolites change, but exactly where and in which cellular contexts these coupled alterations most effectively orchestrate drug action.

Based on this rationale, we designed an integrated analytical strategy to investigate Sol intervention in MPTP-induced PD-like pathology. It should be noted that human idiopathic PD represents a chronic, decades-long neurodegenerative process characterized by protein aggregation; the acute MPTP model serves as an accelerated, high-fidelity mimic of the downstream mitochondrial complex I failure and selective, irreversible loss of the dopaminergic neuron population. By inducing genuine neurotoxic death rather than transient functional inactivation, this model provides a highly stringent platform for evaluating the in situ neuroprotective and homeostatic rescue capacity of Sol against severe bioenergetic collapse [23]. We first used network pharmacology to identify candidate targets shared by Sol and PD, then combined brain single-cell eQTL-based Mendelian randomization, single-cell transcriptomic expression localization, and single-cell virtual knockout analysis to prioritize cell-type-specific regulatory nodes. We next established an MPTP mouse model and evaluated the effects of Sol on behavioral abnormalities. Finally, AFADESI-MSI-based spatial metabolomics, pathway enrichment, gene–metabolite co-enrichment, and in situ metabolite imaging were used to connect candidate targets with region-specific metabolic remodeling. This workflow was designed to move from target prediction to cell-type-aware functional prioritization and spatial metabolic validation.

## 2. Materials and Methods

### 2.1. Drugs and Reagents

Sol was obtained from Aladdin (Shanghai Aladdin Biochemical Technology Co., Ltd., Shanghai, China; catalog No. S107395-25g; CAS No. 13190-97-1). MPTP hydrochloride was obtained from MedChemExpress (MedChemExpress LLC, Monmouth Junction, NJ, USA; catalog No. HY-W114750; CAS No. 28289-54-5). L-DOPA was obtained from Aladdin (catalog No. D111048-100g; CAS No. 59-92-7). Chromatographic-grade methanol, acetonitrile, and formic acid were purchased from Thermo Fisher Scientific (Fisher Scientific Worldwide (Shanghai) Co., Ltd., Shanghai, China). Deionized water was prepared using a Milli-Q Academic ultrapure water system (Millipore, Bedford, MA, USA). CMC-Na embedding agent and other routine reagents were analytical grade (Aladdin).

The vehicle consisted of 2% DMSO, 30% PEG300, 2% Tween 80, and 66% sterile normal saline were purchased from Sigma-Aldrich (St. Louis, MO, USA). For Sol preparation, the vehicle was mixed under sterile warm conditions to assist solubilization and to ensure complete dissolution before administration.

### 2.2. Physicochemical Prediction, Target Collection, and Network Pharmacology

The two-dimensional structure, molecular formula, and standardized chemical information of Sol were obtained from PubChem (https://pubchem.ncbi.nlm.nih.gov, accessed on 22 July 2026) [24]. SwissADME (http://www.swissadme.ch, accessed on 22 July 2026) [22] and ADMETlab 3.0 (https://admetlab3.scbdd.com, accessed on 22 July 2026) [25] were used to predict drug-likeness and pharmacokinetic properties, including lipophilicity, gastrointestinal absorption, blood–brain barrier permeability, plasma protein binding, Caco-2 permeability, P-glycoprotein substrate status, LogS, LogD, LogP, hERG risk, and human intestinal absorption.

Candidate Sol targets were collected from SwissTargetPrediction (http://www.swisstargetprediction.ch, accessed on 22 July 2026), SuperPred (https://prediction.charite.de, accessed on 22 July 2026), SEA (http://sea.bkslab.org, accessed on 22 July 2026), and PharmMapper (v2017, https://bio.tools/pharmmapper-drug, accessed on 22 July 2026) [26,27,28,29]. PD-related disease genes were collected from GeneCards (https://www.genecards.org, accessed on 22 July 2026), OMIM (https://www.omim.org, accessed on 22 July 2026), DisGeNET(v8.0, https://www.disgenet.org, accessed on 22 July 2026), and TTD(v4.3.02, https://db.idrblab.net/ttd, accessed on 22 July 2026) [30,31,32,33]. All target names were standardized to official gene symbols, merged, and deduplicated. Sol-related targets and PD-related genes were intersected to obtain candidate targets shared by Sol and PD. Gene Ontology (GO; biological process, cellular component, and molecular function) and KEGG pathway enrichment analyses were performed in R version 4.3.2 using clusterProfiler and org.Hs.eg.db [34], and representative results were visualized using ggplot2, enrichplot, ggVennDiagram/VennDiagram, dplyr, tidyr, and related tidyverse packages.

### 2.3. Single-Cell eQTL-Based Mendelian Randomization

To improve the genetic and cell-type prioritization of candidate targets, the overlapping Sol-PD candidate genes were converted to Ensembl IDs and matched to SingleBrain single-cell eQTL summary statistics (v1.0, https://singlebrain.nygenome.org, accessed on 22 July 2026) [35]. The SingleBrain resource covers major brain cell types, including neurons, astrocytes, oligodendrocytes, oligodendrocyte precursor cells (OPCs), microglia, and vascular-associated cells. The PD outcome GWAS dataset was obtained from the OpenGWAS database (ebi-a-GCST90018894, https://gwas.mrcieu.ac.uk, accessed on 22 July 2026), which indexes large-scale PD GWAS summary statistics [36].

Allele harmonization and Mendelian randomization analyses were conducted using the TwoSampleMR workflow in R version 4.3.2 [37], with supporting packages including ieugwasr, data.table, dplyr, ggplot2, and forest-plot visualization tools. When a single instrumental variant was available, the Wald ratio method was used; when multiple variants were available, the inverse-variance weighted method was used as the primary analysis. Odds ratios (ORs), 95% confidence intervals (CIs), and *p*-values were used to evaluate the direction and strength of association between candidate gene expression in each cell type and PD risk. Associations with *p* < 0.05 were considered nominally significant, and FDR correction was used to highlight higher-confidence signals.

### 2.4. Single-Cell Transcriptome Processing and Candidate-Gene Localization

Single-cell expression localization was performed using the public brain single-cell transcriptomic dataset GSE243639 [38]. Cell-type annotations were obtained from the annotation table provided by the GEO dataset authors and were checked for consistency with canonical marker expression. The main annotated cell populations included oligodendrocytes, astrocytes, microglia, OPCs, neurons, vascular-associated cells, and T cells.

Seurat was used for quality control, normalization, highly variable gene selection, scaling, principal component analysis, neighbor graph construction, clustering, and UMAP visualization. Candidate-gene expression patterns were visualized with FeaturePlot and related Seurat functions. The R packages used for single-cell processing and visualization included Seurat [39], patchwork, ggplot2, dplyr, and related tidyverse packages.

### 2.5. Single-Cell Virtual Knockout Analysis

To further evaluate the potential functional consequences of prioritized candidate genes in disease-state cellular contexts, single-cell virtual knockout analysis was performed using scTenifoldKnk (v1.0.3, https://github.com/cailab-tamu/scTenifoldKnk, accessed on 22 July 2026) [40] on PD-state cells from the GSE243639 single-cell transcriptomic dataset. Candidate genes were tested in the cell populations where they showed detectable expression and/or cell-type relevance in the preceding scMR and FeaturePlot analyses, including neurons, astrocytes, oligodendrocytes, OPCs, and vascular-associated cells.

For each gene–cell type pair, a cell-type-specific gene regulatory network was inferred from normalized single-cell expression profiles, and the target gene was computationally removed by setting its outgoing regulatory edges to zero. The perturbed network was compared with the original network to estimate downstream transcriptional perturbation. Genes with adjusted *p* < 0.05 were considered significantly perturbed. Volcano plots displayed log10-transformed perturbation distance against −log10 adjusted *p*-value, with the knocked-out target gene marked separately. Rank-ordered perturbation signatures were then subjected to gene set enrichment analysis (GSEA) [41] using Gene Ontology biological-process terms, and representative enriched terms were visualized with ridgeplots. Because the analysis is computational, virtual knockout results were interpreted as functional-prioritization evidence rather than direct experimental proof of target engagement. The virtual knockout analysis in Section 3.6 does not cover all cell types present in the SingleBrain database (Section 2.3) but instead focuses on gene–cell type combinations prioritized jointly by scMR, single-cell expression localization, and cell-type relevance.

### 2.6. Animal Grouping and MPTP Model Establishment

Male C57BL/6 mice aged 8–10 weeks were purchased from Charles River Laboratories (Beijing, China) and randomly assigned to four groups (*n* = 6 per group, for a total of 24 animals): Control, MPTP, Sol + MPTP, and L-DOPA + MPTP. Randomization was used to allocate experimental units to these groups. The randomization sequence was generated using the standard random number generator in Microsoft Excel. Mice were housed under specific pathogen-free conditions at 24 ± 2 °C and 60% ± 5% humidity under a 12 h light/dark cycle, with food and water available ad libitum. Potential confounders were controlled through a structured experimental design. Group allocation and the sequence of treatments/measurements were fully randomized to prevent order-related bias. To mitigate environmental variance, all cages were maintained under identical, strictly controlled laboratory conditions (constant temperature, humidity, and a 12 h light/dark cycle) on the same shelf levels of the housing racks. Additionally, the order of treatments, sample collections, and subsequent measurements was fully randomized across all groups to eliminate time-of-day effects or batch bias. All assessments were conducted by investigators blinded to the group allocations. Only male mice were used to reduce variability associated with sex-hormone cycling in behavioral and neurochemical readouts and to maintain consistency in this mechanism-focused evaluation. All animal procedures were approved by the Laboratory Animal Management and Ethics Committee of the China National Tobacco Quality Supervision and Test Center (Approval No.: CTQTC-SYXK-2025001). During the treatment process, we used high-quality, fine-sized needles for intraperitoneal injections to reduce acute tissue damage.

The overall experiment lasted 34 days and included 7 days of acclimation, 7 days of pretreatment (our study was intentionally designed around a neuroprotective/prophylactic paradigm rather than a post-damage therapeutic rescue model), 7 days of drug intervention combined with MPTP model induction, 8 days of behavioral testing, and 5 days of tissue collection and AFADESI-MSI analysis. MPTP was administered at 30 mg/kg once daily by intraperitoneal injection [42]. Sol was administered at 10 mg/kg once daily, and L-DOPA was administered at 4 mg/kg once daily as the positive control. During the model-induction phase, Sol or L-DOPA was administered first, followed by MPTP injection 1–2 h later. Control and MPTP mice received equal volumes of the corresponding vehicle where appropriate. All six mice in each group underwent behavioral testing, and brain tissues were collected for downstream spatial metabolomic analysis (*n* = 3) after behavioral assessment. This MPTP model was selected because it is a well-established toxin-based model for dopaminergic injury and PD-like motor deficits [42,43].

### 2.7. Behavioral Assessment

Behavioral tests (*n* = 6) were performed after the drug-treatment and model-induction procedures to evaluate motor coordination, spontaneous locomotor activity, exploratory behavior, anxiety-like behavior, and spatial working memory. All tests were conducted in a quiet, dimly lit room between 09:00 and 15:00. The apparatus was cleaned with 75% ethanol between trials to eliminate olfactory cues. In order to minimize the suffering and discomfort of the animals, we incorporated strict ethical prevention measures into our experimental plan. In the behavioral tests, we strictly controlled the stress factors in the environment (such as strong light and sudden noises) and allowed the animals to rest quietly between each test.

Open field test: Spontaneous locomotor activity and exploratory/anxiety-like behavior were assessed in a square arena (100 cm × 100 cm × 50 cm). Each mouse was placed in the center of the arena and allowed to explore freely for 10 min. Locomotor trajectories were recorded and analyzed using VisuTrack software (v1.0, Shanghai Xinruan Information Technology Co., Ltd., Shanghai, China). Total distance traveled was used to evaluate locomotor activity, and the proportion of time spent in the center zone was used as an index of exploratory or anxiety-like behavior.

Y-maze test: Spatial working memory was evaluated using the spontaneous alternation task. The maze consisted of three identical arms (labeled A, B, and C; 50 cm × 10 cm × 20 cm) diverging at 120° angles. Each mouse was placed at the end of one arm and allowed to explore freely for 10 min. An alternation was defined as consecutive entries into all three arms (for example, ABC, BCA, or CAB). The spontaneous alternation rate (SAR) was calculated as: SAR (%) = number of alternations/(total arm entries − 2) × 100.

Rotarod test: Motor coordination and balance were assessed using a rotarod apparatus (Shanghai Xinruan Information Technology Co., Ltd., Shanghai, China). Before testing, mice were trained for 3 consecutive days to adapt to the rotating rod. On the test day, the rod accelerated from 4 to 40 rpm over 5 min. The latency to fall was recorded for each mouse, and the average of three trials was used for statistical analysis.

### 2.8. Tissue Collection and Section Preparation

After behavioral assessment, mice were subjected to terminal anesthesia with 2.5% tribromoethanol (250 mg/kg, intraperitoneally; T48402, Sigma-Aldrich, St. Louis, MO, USA) before tissue collection. This anesthetic was used only for terminal tissue harvesting. After deep anesthesia was confirmed by loss of the pedal reflex, mice were euthanized by decapitation, and whole brains were rapidly excised on an ice-cold plate and weighed.

To preserve tissue morphology and metabolite localization, brain tissues were flash-frozen in isopentane chilled with liquid nitrogen and stored at −80 °C until AFADESI-MSI analysis. For MSI experiments, brain samples were removed from −80 °C storage and equilibrated at −20 °C for 6 h. Sagittal sections of 10 µm thickness were prepared using a cryostat (Leica Biosystems Nussloch GmbH, Baden-Württemberg, Germany), mounted directly onto positively charged microscope slides, vacuum-dried for 15 min, and then subjected to AFADESI-MSI analysis.

### 2.9. AFADESI-MSI Analysis

Mass spectrometry imaging was performed using an AFADESI-MSI platform (Beijing Viktor Technology Co., Ltd., Beijing, China) coupled to a Q Exactive mass spectrometer (Thermo Fisher Scientific, Waltham, MA, USA). The nebulization solvent was acetonitrile/water (8:2, *v*/*v*) at a flow rate of 6 µL/min. The nebulization voltage was set to ±3.0 kV, and the auxiliary gas pressure was maintained at 0.7 MPa.

Data were acquired (*n* = 3) in both positive and negative ion modes over a mass range of *m*/*z* 100–1000 at a resolution of 120,000. Imaging was conducted using continuous raster scanning with a scanning speed of 0.2 mm/s and a line spacing of 0.2 mm. The vertical distance between the nebulizer needle and tissue surface was approximately 3 mm, the nebulization angle was 60°, and the ion transfer tube temperature was 350 °C.

### 2.10. Spatial Metabolomics Data Processing and Metabolite Annotation

Raw MSI data files were converted to .cdf format using Xcalibur software (v2.2, Thermo Fisher Scientific) and imported into MassImager software (v1.0, Beijing Viktor Technology Co., Ltd.) for background subtraction, normalization, and ion-image reconstruction. Whole-brain (ALL) and striatal (STR) regions (*n* = 3) of interest were manually delineated in MassImager according to tissue morphology and adjacent hematoxylin-eosin-stained sections, with reference to a mouse brain atlas. For each metabolic feature, the mean normalized ion intensity within each ROI was calculated for each animal and used as the statistical unit for downstream analysis.

Features were processed separately for four datasets: whole-brain positive-ion mode (ALL_pos), whole-brain negative-ion mode (ALL_neg), striatal positive-ion mode (STR_pos), and striatal negative-ion mode (STR_neg). The AFADESI-MSI workflow provided a spatial resolution of approximately 100 µm and enabled sensitive detection of small molecules across both ion modes within *m*/*z* 100–1000. Metabolites were annotated solely by accurate mass matching against HMDB and METLIN [44,45] with a mass error threshold of <5 ppm.

### 2.11. Pathway Enrichment and Gene–Metabolite Joint Enrichment

Differential metabolic features were screened using univariate *p* < 0.05, with VIP > 1.0 used where multivariate model-based screening was applicable. Pathway enrichment of differential metabolites was performed using the pathway analysis module of MetaboAnalyst 6.0 [46], which combines over-representation analysis with pathway topology analysis. Pathways with raw *p* < 0.05 and pathway impact > 0.10 were considered enriched. Semi-quantitative bar plots accompanying MSI ion images were generated from ROI-averaged normalized intensities for each animal.

Candidate genes and differential metabolites were jointly analyzed using IMPaLA (v13, http://impala.molgen.mpg.de/impala, accessed on 22 July 2026) [47] to identify pathways supported by both gene-level and metabolite-level evidence. Enriched pathways were further organized into functional modules, including membrane lipid remodeling, lipid-derived kinase signaling, stress/inflammation/hypoxia responses, and neuronal disease-associated pathways. Visualization was performed in R version 4.3.2 using ggplot2, ggpubr, ropls, ComplexUpset/UpSetR, ggalluvial, circlize, dplyr, tidyr, readr, stringr, and related packages.

### 2.12. Statistical Analysis

Statistical analyses were performed using R version 4.3.2 and GraphPad Prism8.0. Behavioral outcomes and ROI-averaged normalized ion intensities are presented as mean ± SEM. For comparisons among multiple groups, one-way analysis of variance followed by Tukey’s post hoc test was used when data met parametric assumptions; otherwise, nonparametric tests were applied. For pairwise comparisons, normality was assessed before applying Student’s *t*-test or the Mann–Whitney U test.

PLS-DA and related multivariate analyses were conducted using the ropls package (v1.43.0) [48] to evaluate metabolic separation among Control, MPTP, and Sol groups in different ROI and ion-mode datasets. Model robustness was evaluated by 5-fold cross-validation and 200 permutation tests where appropriate. UpSet plots, bubble plots, bar plots, Sankey diagrams, chord diagrams, and feature distribution plots were generated using the R packages described above. *p* < 0.05 was considered statistically significant, with * *p* < 0.05, ** *p* < 0.01, and *** *p* < 0.001 used in the figures.

Due to the nature of the interventions, complete blinding during the entire study was not feasible. However, blinding was maintained during the critical evaluation stages.

During allocation & conduct of the experiment: The primary investigators were aware of the group allocations to ensure the correct administration of treatments and procedural accuracy.

During outcome assessment: The researchers performing the outcome assessments (e.g., mass spectrometry imaging, molecular assays, data quantification) were blinded to the treatment groups to eliminate subjective bias.

During data analysis: Data analysts were blinded to the group identities, working with anonymized datasets until the statistical evaluation was completed.

## 3. Results

### 3.1. Network Pharmacology Integrated with Single-Cell Mendelian Randomization Prioritizes Candidate Targets of Sol in PD

**Figure 1:**
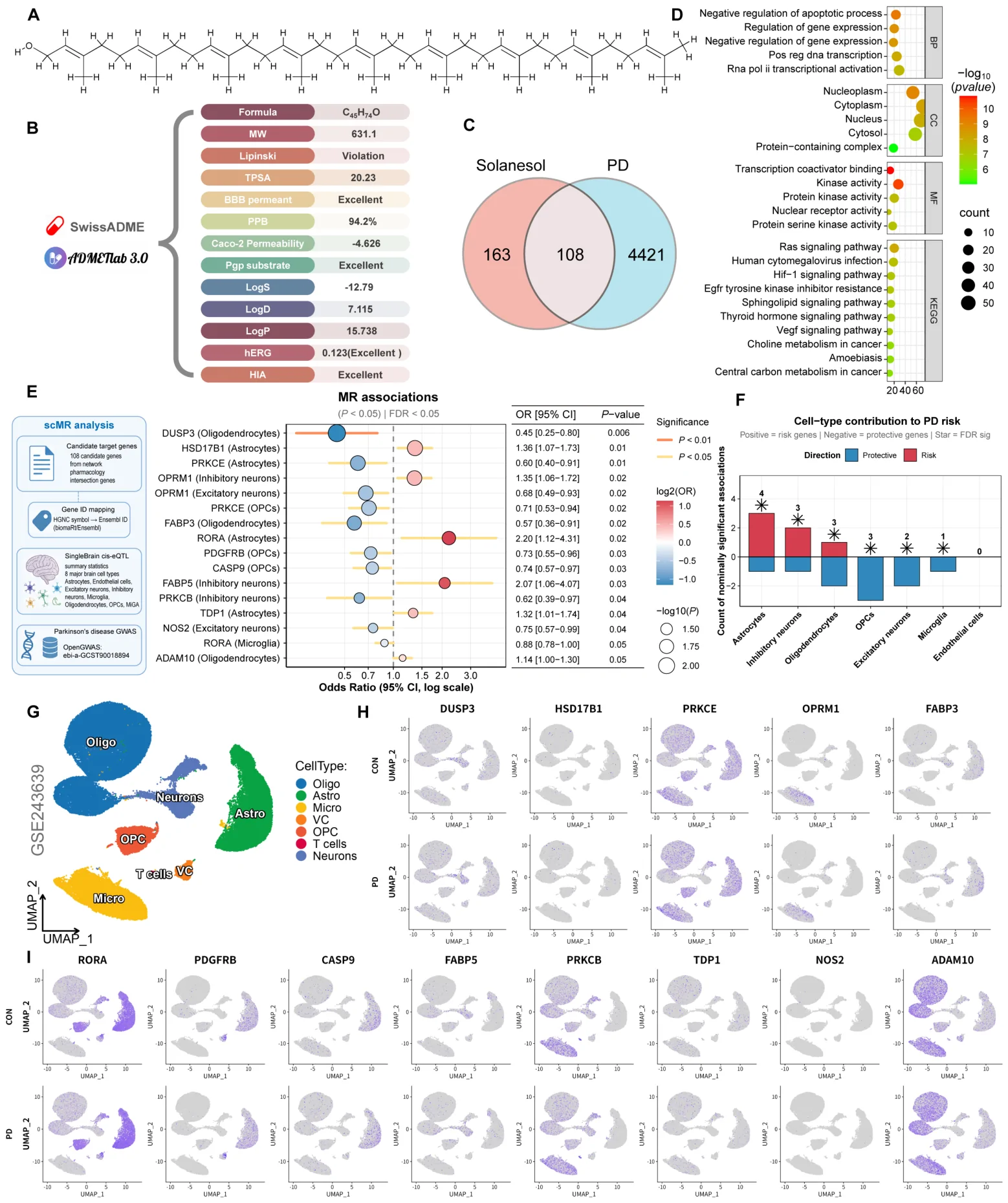
Network pharmacology, single-cell Mendelian randomization, and single-cell expression localization jointly prioritize candidate targets of Sol in PD. (**A**) Two-dimensional chemical structure of Sol. (**B**) Physicochemical and pharmacokinetic properties predicted by SwissADME and ADMETlab 3.0, including molecular formula, molecular weight, TPSA, blood–brain barrier permeability, plasma protein binding, Caco-2 permeability, P-glycoprotein substrate prediction, LogS, LogD, LogP, hERG risk, and human intestinal absorption. (**C**) Venn intersection of Sol-related targets and PD-related disease genes, yielding 108 overlapping targets. (**D**) GO and KEGG enrichment analyses of the 108 overlapping targets. Bubble size indicates gene count, and color indicates −log10(*p*-value). (**E**) scMR workflow and significant candidate-gene associations. The left panel shows candidate target input, gene ID mapping, SingleBrain single-cell eQTL integration, and PD GWAS outcome data; the forest plot shows MR associations between candidate genes and PD risk across cell types. (**F**) Number of nominally significant MR associations across cell types. Red indicates risk direction, blue indicates protective direction, and asterisks indicate FDR-significant associations. (**G**) UMAP clustering and major cell-type annotations. (**H**,**I**) FeaturePlot visualization of candidate-gene expression in different cell populations under CON and PD states. Abbreviations: Oligo, oligodendrocytes; Astro, astrocytes; Micro, microglia; OPC, oligodendrocyte precursor cells; VC, vascular cells.

To investigate the potential molecular basis by which Sol improves PD-like pathology, we first constructed a multi-layer screening workflow integrating physicochemical prediction, disease-target intersection, pathway enrichment, scMR, and single-cell expression localization. Sol has a long-chain lipid-like structure, a molecular formula of C45H74O, and a predicted molecular weight of 631.1. SwissADME and ADMETlab 3.0 predictions indicated high lipophilicity, favorable gastrointestinal absorption, blood–brain barrier permeability, and plasma protein binding, supporting its potential ability to influence the central lipid microenvironment (Figure 1A,B).

By intersecting Sol-related candidate targets with PD-related disease genes, we obtained 108 overlapping targets (Figure 1C). Functional enrichment showed that these targets were associated with negative regulation of apoptosis, gene-expression regulation, protein kinase activity, nuclear receptor activity, and transcriptional regulation. KEGG enrichment further highlighted Ras signaling, HIF-1 signaling, sphingolipid signaling, VEGF signaling, and choline metabolism (Figure 1D), suggesting that Sol may act through coordinated lipid signaling, kinase signaling, inflammatory stress, and cell-survival networks rather than through a single target.

To refine target prioritization at the genetic and cell-type levels, the 108 overlapping targets were integrated with brain single-cell eQTL data and PD GWAS data for scMR analysis (Figure 1E). Several candidate genes showed significant or marginal associations with PD risk in oligodendrocytes, astrocytes, OPCs, excitatory neurons, and inhibitory neurons. DUSP3, HSD17B1, PRKCE, OPRM1, FABP3, RORA (Astrocytes, Microglia), PDGFRB, CASP9, FABP5, PRKCB, TDP1, NOS2, and ADAM10 displayed cell-type-dependent genetic association signals with different effect directions, indicating that Sol-related candidate targets may participate in PD risk through cell-type-specific mechanisms (Figure 1E,F).

Single-cell transcriptomic localization further mapped these candidate genes to major brain cell populations, including oligodendrocytes, astrocytes, microglia, OPCs, neurons, vascular-associated cells, and T cells (Figure 1G). Feature Plot visualization showed broad expression of PRKCE, PRKCB, and ADAM10 across multiple cell populations, whereas FABP3, FABP5, RORA, PDGFRB, and NOS2 showed more pronounced cell-population preferences (Figure 1H,I). Together, network pharmacology, scMR, and expression localization prioritized lipid transport, kinase signaling, inflammatory stress, and cell-death-related genes for subsequent virtual knockout analysis and spatial metabolomic validation.

### 3.2. Sol Improves MPTP-Induced Behavioral Abnormalities and Reshapes Neurotransmitter-Related Spatial Distributions

**Figure 2:**
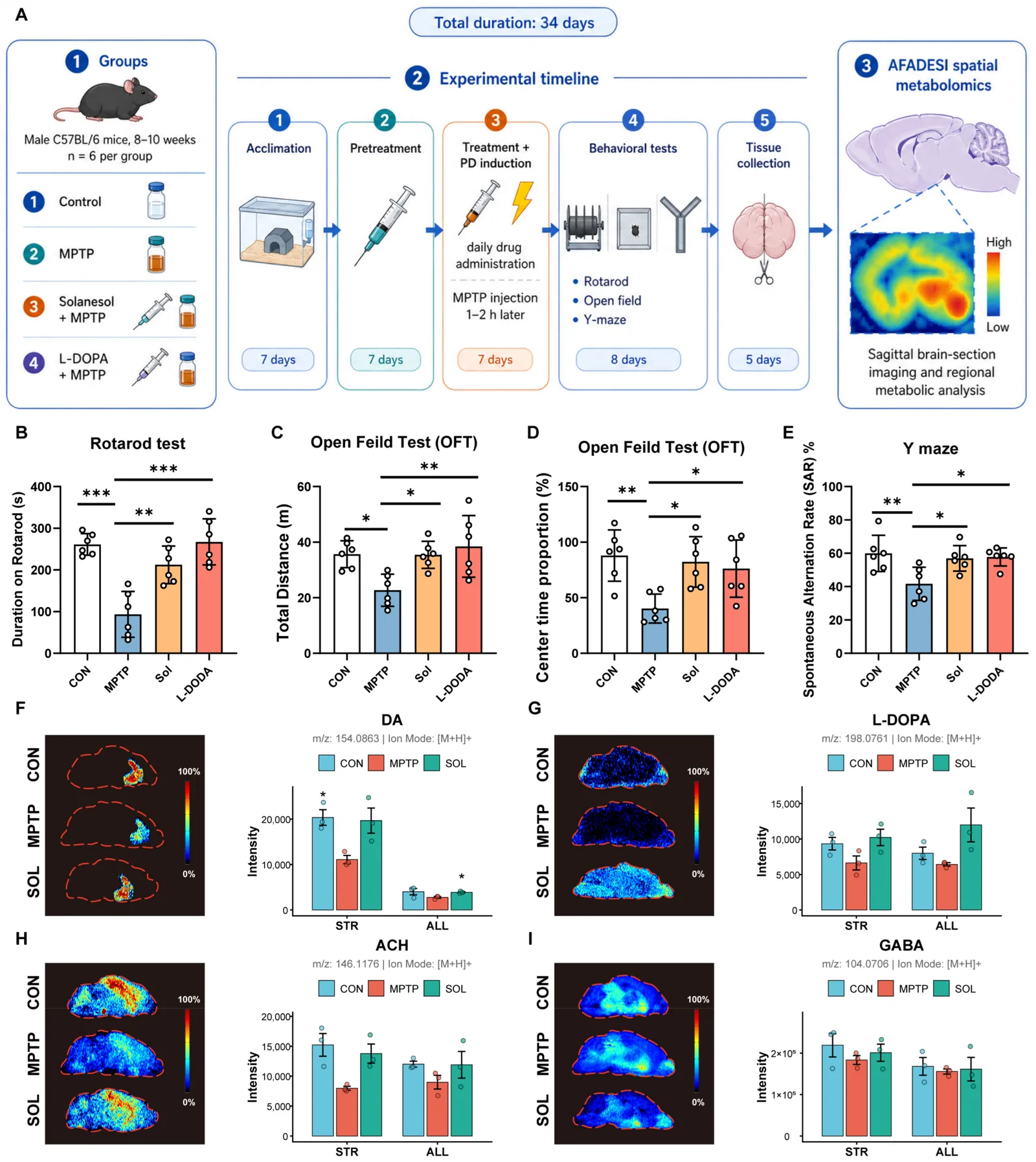
Sol improves MPTP-induced behavioral abnormalities and reshapes neurotransmitter-related spatial distributions. (**A**) Experimental design. Male C57BL/6 mice were randomly assigned to Control, MPTP, Sol + MPTP, and L-DOPA + MPTP groups (*n* = 6 per group). The experiment lasted 34 days, including acclimation, pretreatment, drug intervention combined with MPTP induction, behavioral testing, tissue collection, and AFADESI-MSI analysis. MPTP, Sol, and L-DOPA were administered at 30, 10, and 4 mg/kg once daily, respectively. (**B**) Rotarod test. (**C**,**D**) Open field test, including total distance and center-time proportion. (**E**) Y-maze spontaneous alternation rate. (**F**) AFADESI-MSI imaging and ROI quantification of DA, *m*/*z* 154.0863, [M + H]^+^. (**G**) L-DOPA, *m*/*z* 198.0761, [M + H]^+^. (**H**) Acetylcholine, *m*/*z* 146.1176, [M + H]^+^. (**I**) GABA, *m*/*z* 104.0706, [M + H]^+^ [49,50]. Red dashed lines indicate brain-tissue boundaries; color scales indicate relative ion intensity. Bar plots are shown as mean ± SEM; * *p* < 0.05, ** *p* < 0.01, *** *p* < 0.001.

Based on the computational screening results, we established an MPTP-induced mouse model and included Control, MPTP, Sol + MPTP, and L-DOPA + MPTP groups. The 34-day experimental timeline consisted of acclimation, pretreatment, drug intervention combined with MPTP induction, behavioral assessment, tissue collection, and AFADESI-MSI analysis (Figure 2A). This design allowed Sol exposure during the injury-formation phase and enabled spatial metabolic assessment after behavioral phenotypes were established. The results show MPTP markedly reduced rotarod latency, indicating impaired motor coordination and balance. Sol significantly prolonged rotarod latency, with an improvement trend approaching that observed in the L-DOPA group (Figure 2B). In the open field test, MPTP reduced both total distance traveled and center-zone time proportion, reflecting impaired locomotor activity and exploratory behavior. Sol restored both indices (Figure 2C,D). In the Y-maze test, MPTP reduced spontaneous alternation, whereas Sol and L-DOPA increased the alternation rate, suggesting improvement of MPTP-induced spatial working-memory or exploratory abnormalities (Figure 2E).

We next used AFADESI-MSI to connect behavioral improvement with spatial changes in neurotransmitter-related small molecules. Dopamine (DA) was decreased in MPTP-treated mice, especially in the striatum, whereas Sol restored DA signal intensity (Figure 2F). L-DOPA spatial signal was also reduced in the MPTP group and increased after Sol treatment, suggesting partial restoration of the dopaminergic precursor environment (Figure 2G). Acetylcholine decreased after MPTP exposure but showed recovery in the striatum and whole-brain ROI after Sol treatment (Figure 2H). In contrast, GABA showed weaker spatial and quantitative changes, indicating that Sol may preferentially influence dopaminergic and cholinergic metabolic axes in this model (Figure 2I). Dopamine, levodopa, acetylcholine, and GABA are neurotransmitters that have been extensively investigated by our group and other researchers. In this study, their identities were confirmed based on their accurate MS1 masses and their characteristic distribution patterns across different brain regions in brain tissue [49,50].

### 3.3. Whole-Brain and Striatal Spatial Metabolomics Reveal Regional Metabolic Reprogramming Induced by Sol

**Figure 3:**
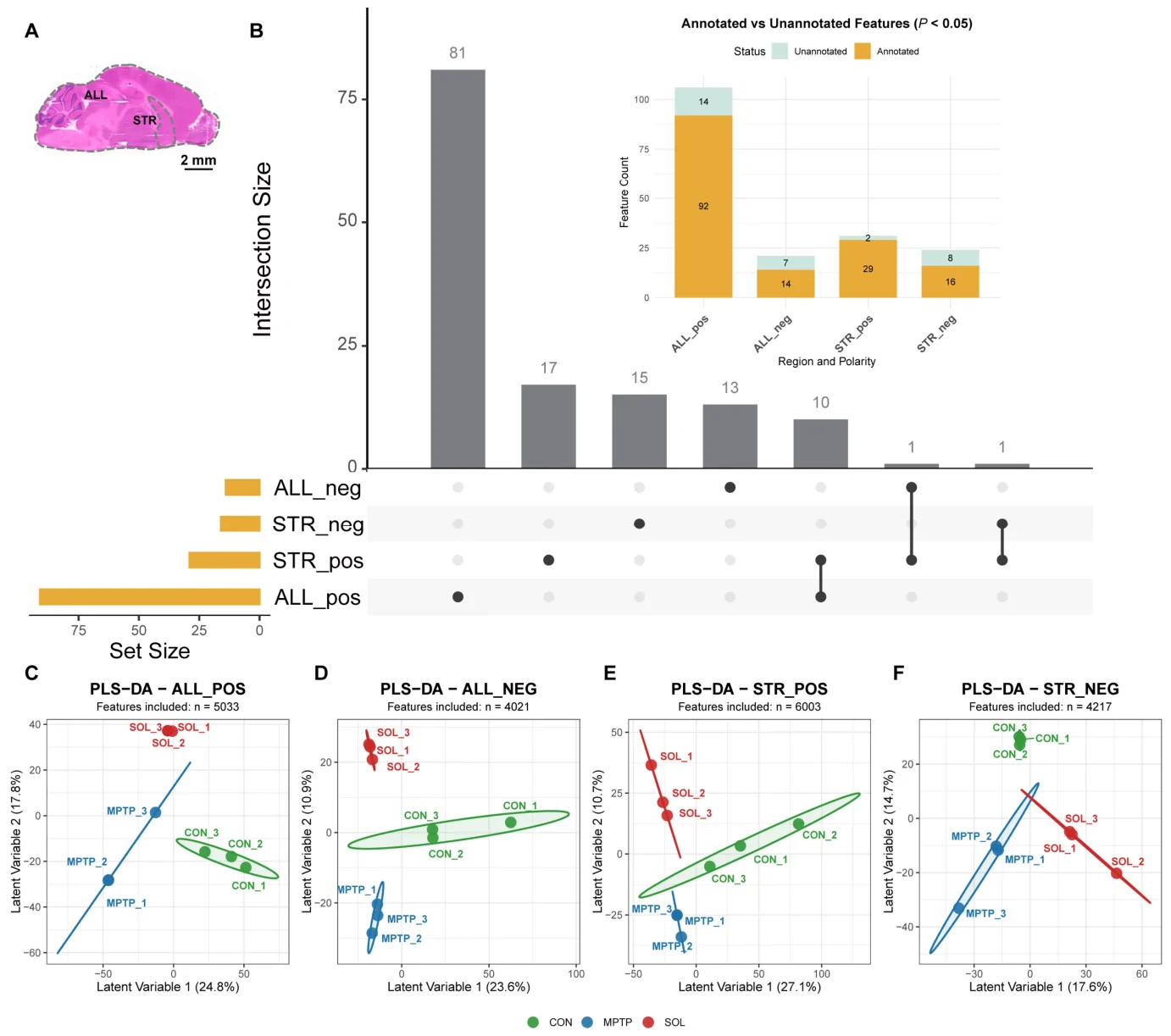
Whole-brain and striatal spatial metabolomics reveal regional metabolic changes after MPTP injury and Sol intervention. (**A**) Delineation of whole-brain (ALL) and striatal (STR) ROIs on sagittal brain sections. (**B**) UpSet analysis of differential metabolic features across ROI/ion-mode datasets and annotated/unannotated feature statistics (Note: The annotations in this section are based on MS1-level mass spectrometric information). ALL_pos, whole-brain positive-ion mode; ALL_neg, whole-brain negative-ion mode; STR_pos, striatal positive-ion mode; STR_neg, striatal negative-ion mode. (**C**) PLS-DA of ALL_pos features (*n* = 5033). (**D**) PLS-DA of ALL_neg features (*n* = 4021). (**E**) PLS-DA of STR_pos features (*n* = 6003). (**F**) PLS-DA of STR_neg features (*n* = 4217). Green, blue, and red indicate CON, MPTP, and SOL groups, respectively.

To further characterize spatial metabolic alterations after Sol intervention, we delineated whole-brain and striatal ROIs on sagittal brain sections and extracted metabolic features in positive and negative ion modes (Figure 3A). The largest number of differential features was detected in the whole-brain positive-ion dataset, with 92 annotated and 14 unannotated features. The whole-brain negative-ion dataset contained 14 annotated and 7 unannotated features; the striatal positive-ion dataset contained 29 annotated and 2 unannotated features; and the striatal negative-ion dataset contained 16 annotated and 8 unannotated features (Figure 3B). The annotation results presented in this section are based on MS1 (first-level mass spectrometry) information. These results indicate that the whole-brain ROI captures broad metabolic disturbances, whereas the striatal ROI provides local information more directly related to dopaminergic injury. UpSet analysis showed both shared and region- or polarity-specific differential features across the four ROI/ion-mode datasets (Figure 3B). The whole-brain positive-ion mode contained the largest set of dataset-specific differential metabolites, whereas both positive and negative striatal modes retained independent region-specific signals. Thus, MPTP injury and Sol intervention generated spatially heterogeneous metabolic changes that cannot be fully represented by a single ROI or ion mode.

PLS-DA further confirmed group discrimination in whole-brain positive, whole-brain negative, striatal positive, and striatal negative datasets (Figure 3C–F). Control, MPTP, and Sol groups formed relatively clear clusters. The R^2^X, R^2^Y, and Q^2^Y values for all groups in Figure 3C met the required thresholds, and the detailed information is provided in Supplementary Information S1. The separation between MPTP and Control indicated stable metabolic disturbance after model induction, whereas the Sol group formed a distinct metabolic state and partially shifted toward the Control group in several dimensions. These results suggest that Sol does not simply reverse a single metabolite change but induces region-selective metabolic network reorganization (Supporting Information, Table S1).

### 3.4. Candidate Genes and Spatially Differential Metabolites Converge on Lipid Metabolism, Inflammatory Stress, and Kinase Signaling

**Figure 4:**
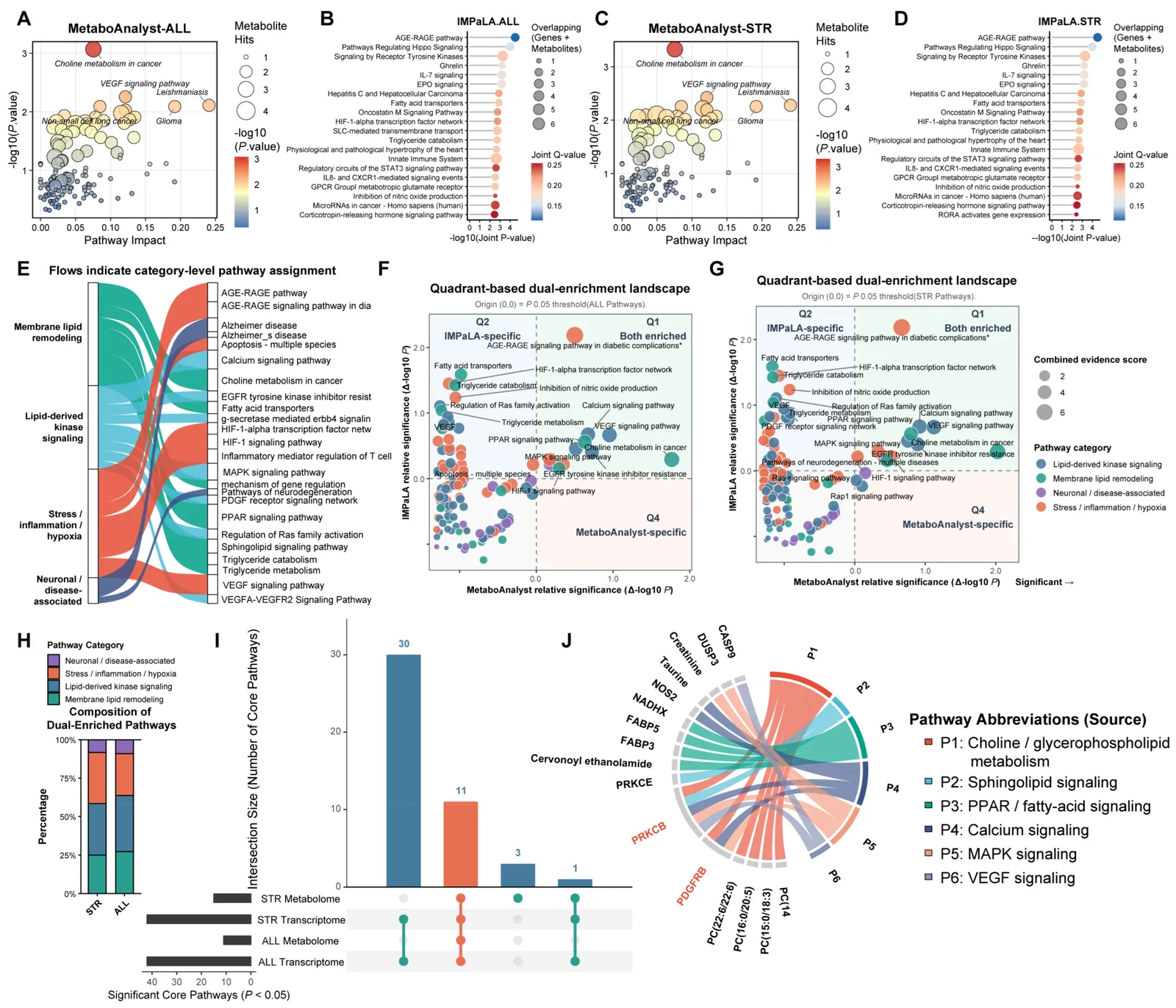
Candidate genes and spatially differential metabolites co-enrich in lipid metabolism, inflammatory stress, and kinase-signaling networks. (**A**) MetaboAnalyst pathway enrichment of whole-brain differential metabolites. (**B**) IMPaLA joint enrichment of whole-brain differential metabolites and candidate genes. (**C**) MetaboAnalyst pathway enrichment of striatal differential metabolites. (**D**) IMPaLA joint enrichment of striatal differential metabolites and candidate genes. (**E**) Sankey diagram assigning enriched pathways to membrane lipid remodeling, lipid-derived kinase signaling, stress/inflammation/hypoxia responses, and neuronal disease-associated pathways. (**F**,**G**) Quadrant-based dual-enrichment landscapes for whole-brain and striatal analyses. Q1, jointly enriched; Q2, IMPaLA-specific; Q4, MetaboAnalyst-specific. * *p* < 0.05. (**H**) Functional category composition of shared enriched pathways. (**I**) UpSet analysis of core pathways across spatial metabolomic and candidate-gene enrichment layers. (**J**) Chord plot linking core metabolites, candidate genes, and six key pathways. P1, choline/glycerophospholipid metabolism; P2, sphingolipid signaling; P3, PPAR/fatty-acid signaling; P4, calcium signaling; P5, MAPK signaling; P6, VEGF signaling.

To mechanistically integrate candidate genes with spatial metabolomics, differential metabolites from whole-brain and striatal datasets were analyzed using MetaboAnalyst, and candidate genes and differential metabolites were jointly analyzed using IMPaLA. Whole-brain metabolite enrichment highlighted choline metabolism, VEGF signaling, lipid-related signaling, and neurological disease-related pathways in high-impact regions of the enrichment landscape (Figure 4A). IMPaLA joint enrichment further identified AGE-RAGE signaling, receptor tyrosine kinase signaling, fatty-acid transporters, HIF-1 transcriptional network, triglyceride catabolism, and GPCR-related signaling (Figure 4B). In the striatum, MetaboAnalyst again identified choline metabolism, VEGF signaling, and neurological disease-related pathways with relatively high enrichment significance and pathway impact (Figure 4C). IMPaLA results further strengthened the involvement of fatty-acid transport, HIF-1 signaling, inflammatory stress, and receptor tyrosine kinase-related pathways in local striatal metabolic remodeling (Figure 4D). The convergence between whole-brain and striatal results suggests that the effects of Sol in the MPTP model may converge on lipid metabolic remodeling, stress/inflammatory regulation, and kinase signal transduction.

To organize the enrichment landscape, significant pathways were assigned to four functional modules: membrane lipid remodeling, lipid-derived kinase signaling, stress/inflammation/hypoxia responses, and neuronal disease-associated pathways (Figure 4E). Quadrant-based dual-enrichment maps identified pathways enriched by both MetaboAnalyst and IMPaLA as high-confidence shared pathways, while other pathways were metabolite-specific or joint-enrichment-specific (Figure 4F,G). AGE-RAGE signaling, HIF-1 transcriptional network, fatty-acid transporters, triglyceride catabolism, calcium signaling, VEGF signaling, MAPK signaling, and choline metabolism occupied core positions in the dual-enrichment landscape.

Pathway category analysis showed that shared pathways in both the whole-brain and striatal datasets were dominated by lipid-related and stress/inflammatory modules (Figure 4H). UpSet analysis further demonstrated intersections among core pathways from different spatial levels and omics layers (Figure 4I). Finally, chord-plot visualization linked core metabolites and candidate genes to six key pathway groups. Cervonoyl ethanolamide, phosphatidylcholine species, taurine, NADHX, and creatinine were connected with PRKCE, FABP3, FABP5, NOS2, CASP9, and DUSP3 through choline/glycerophospholipid metabolism, sphingolipid signaling, PPAR/fatty-acid signaling, calcium signaling, MAPK signaling, and VEGF signaling (Figure 4J). These findings connect computational target screening with spatial metabolic evidence and support a lipid–inflammatory-kinase network as a central mechanism of Sol action.

### 3.5. In Situ Imaging of Key Lipids and Pathology-Related Small Molecules Validates Sol-Induced Metabolic Remodeling

**Figure 5:**
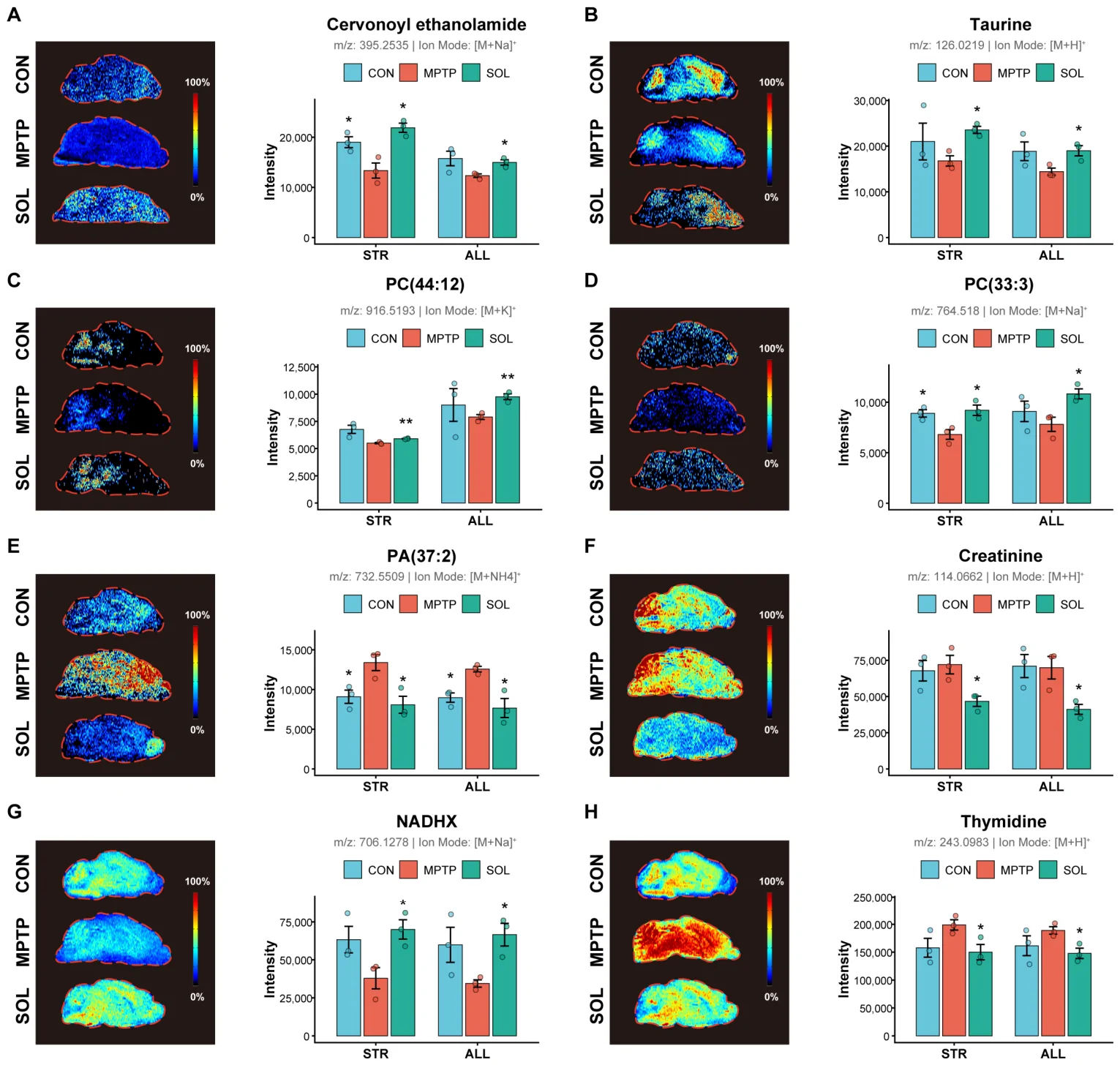
Spatial imaging of key lipids and pathology-related small molecules validates Sol-induced metabolic remodeling. (**A**) Cervonoyl ethanolamide, *m*/*z* 395.2535, [M + Na]^+^. (**B**) Taurine, *m*/*z* 126.0219, [M + H]^+^. (**C**) PC(44:12), *m*/*z* 916.5193, [M + K]^+^. (**D**) PC(33:3), *m*/*z* 764.5181, [M + Na]^+^. (**E**) PA(37:2), *m*/*z* 732.5509, [M + NH_4_]^+^. (**F**) Creatinine, *m*/*z* 114.0662, [M + H]^+^. (**G**) NADHX, *m*/*z* 706.1278, [M + Na]^+^. (**H**) Thymidine, *m*/*z* 243.0983, [M + H]^+^. Representative ion images are shown with ROI-based quantification for the striatum and whole-brain region. Color scales indicate relative ion intensity, and red dashed lines indicate tissue boundaries. Bar plots are shown as mean ± SEM; * *p* < 0.05, ** *p* < 0.01.

Based on the co-enrichment results, we selected representative molecules related to lipid metabolism, redox homeostasis, energy metabolism, and nucleoside metabolism for in situ spatial imaging validation. Cervonoyl ethanolamide decreased in the MPTP group and recovered or increased in the striatum and whole-brain ROI after Sol treatment, suggesting that endogenous lipid signaling molecules may participate in Sol-induced membrane lipid and neuroprotective metabolic regulation (Figure 5A). Taurine decreased after MPTP exposure and increased after Sol treatment, consistent with possible restoration of antioxidant, osmotic, or neuroprotective metabolic processes (Figure 5B). Phospholipid metabolites further supported lipid remodeling as a core outcome. The highly unsaturated phosphatidylcholine PC(44:12) decreased after MPTP exposure but increased in the whole-brain ROI and showed a recovery trend in the striatum after Sol intervention (Figure 5C). PC(33:3) also decreased in the MPTP group and increased after Sol treatment (Figure 5D). Conversely, PA(37:2) increased in the MPTP group and decreased after Sol treatment, indicating that the effect of Sol on lipid metabolism is selective rather than a uniform increase or suppression of all lipid classes (Figure 5E).

Several non-lipid pathology-related molecules also showed clear spatial changes. Creatinine remained relatively high in MPTP-treated mice and decreased after Sol intervention, suggesting altered energy or creatine/creatinine metabolism (Figure 5F). NADHX decreased in the MPTP group and was restored after Sol treatment, pointing to potential repair of NADH/NAD+-related redox homeostasis (Figure 5G). Thymidine increased after MPTP treatment and decreased after Sol intervention, suggesting attenuation of nucleoside metabolism or cell-stress-related processes (Figure 5H). Together, these in situ imaging results validate the lipid metabolic, inflammatory stress, redox, and nucleoside metabolic remodeling axes proposed by the co-enrichment analysis.

### 3.6. Single-Cell Virtual Knockout Analysis Identifies Cell-Type-Specific Regulatory Consequences of Candidate Targets

**Figure 6:**
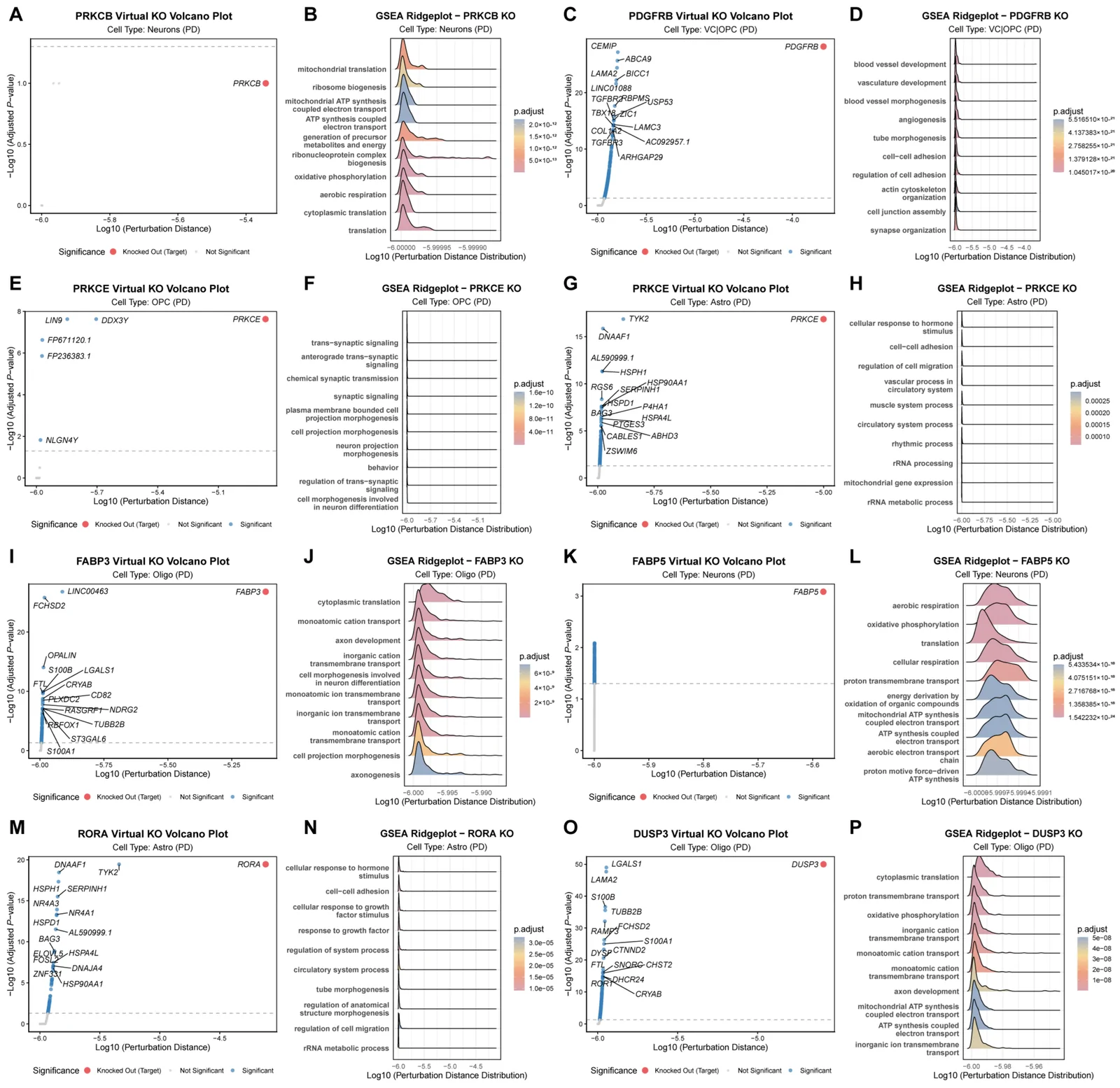
Single-cell virtual knockout analysis identifies cell-type-specific regulatory consequences of candidate targets in PD-state cells. (**A**,**B**) PRKCB virtual knockout in neurons. (**C**,**D**) PDGFRB virtual knockout in VC/OPC cells. (**E**,**F**) PRKCE virtual knockout in OPCs. (**G**,**H**) PRKCE virtual knockout in astrocytes. (**I**,**J**) FABP3 virtual knockout in oligodendrocytes. (**K**,**L**) FABP5 virtual knockout in neurons. (**M**,**N**) RORA virtual knockout in astrocytes. (**O**,**P**) DUSP3 virtual knockout in oligodendrocytes. In volcano plots, the red point indicates the knocked-out target gene, blue points indicate significantly perturbed genes, and gray points indicate non-significant genes; the x-axis shows log10-transformed perturbation distance and the y-axis shows −log10 adjusted *p*-value. Ridgeplots show representative enriched GO biological processes from GSEA of virtual-knockout perturbation signatures. Abbreviations: Astro, astrocytes; Oligo, oligodendrocytes; OPC, oligodendrocyte precursor cells; VC, vascular-associated cells; KO, knockout.

To further move from candidate-gene prioritization toward cell-type-specific functional hypotheses, we performed single-cell virtual knockout analysis in PD-state cell populations. Volcano plots summarized the transcriptional perturbation caused by in silico deletion of each target, and GSEA ridgeplots organized perturbed genes into biological processes (Figure 6). It should be noted that Figure 6 presents the results of priority virtual knockout, and no microglial cells are included. In PD neurons, PRKCB virtual knockout produced a compact but functionally coherent perturbation signature enriched for mitochondrial translation, ribosome biogenesis, oxidative phosphorylation, aerobic respiration, cytoplasmic translation, and ATP synthesis-coupled electron transport (Figure 6A,B). FABP5 virtual knockout in neurons yielded a related energy-metabolic signature involving aerobic respiration, oxidative phosphorylation, cellular respiration, proton transmembrane transport, and mitochondrial ATP synthesis-coupled electron transport (Figure 6K,L). These findings indicate that neuronal PRKCB and FABP5 may influence mitochondrial respiratory and translational programs, consistent with the recovery of dopaminergic signals and NADHX-related redox metabolism observed by spatial MSI.

Virtual knockout also highlighted glial and vascular-associated regulatory axes. PDGFRB deletion in VC/OPC cells strongly perturbed extracellular matrix and vascular-associated genes, including CEMIP, ABCA9, LAMC1, TGFBR3, and COL1A2, and enriched blood vessel development, vasculature development, angiogenesis, tube morphogenesis, cell–cell adhesion, actin cytoskeleton organization, and cell-junction assembly (Figure 6C,D). PRKCE deletion in OPCs enriched trans-synaptic signaling, chemical synaptic transmission, plasma membrane-bounded cell-projection morphogenesis, neuron-projection morphogenesis, and cell morphogenesis involved in neuronal differentiation (Figure 6E,F), whereas PRKCE deletion in astrocytes perturbed stress-response genes such as TYK2, HSPH1, HSP90AA1, HSPD1, and BAG3 and enriched hormone-response, cell-adhesion, migration, circulatory-system, rRNA-processing, and mitochondrial gene-expression terms (Figure 6G,H). RORA knockout in astrocytes produced a related perturbation landscape involving TYK2, NR4A1/NR4A3, heat-shock genes, hormone/growth-factor response, cell adhesion, tube morphogenesis, migration, and rRNA metabolism (Figure 6M,N).

Finally, lipid-binding and phosphatase nodes showed oligodendrocyte-associated effects. FABP3 knockout in oligodendrocytes perturbed OPALIN, S100B, LGALS1, CRYAB, NDRG2, RASGRF1, TUBB2B, and related genes, with enrichment in cytoplasmic translation, ion transport, axon development, neuronal cell morphogenesis, cell-projection morphogenesis, and axonogenesis (Figure 6I,J). DUSP3 knockout in oligodendrocytes yielded the strongest perturbation landscape, affecting LGALS1, LAMA2, S100B, TUBB2B, FCHSD2, RAMP3, CTNND2, DHCR24, and CRYAB, and enriched cytoplasmic translation, oxidative phosphorylation, proton transmembrane transport, monoatomic cation transport, axon development, and mitochondrial electron transport (Figure 6O,P). Together, the virtual knockout results nominate PRKCB/FABP5-linked neuronal mitochondrial regulation, PDGFRB-linked vascular adhesion and morphogenesis, PRKCE/RORA-linked astrocyte-OPC signaling and stress responses, and FABP3/DUSP3-linked oligodendrocyte axon energy programs as cell-type-specific regulatory mechanisms that connect candidate genes with the lipid–inflammatory and mitochondrial metabolic axes identified by spatial metabolomics.

## 4. Discussion

In the present study, we establish a fundamentally new mechanistic framework for the neuroprotective efficacy of Sol in PD. By integrating single-cell Mendelian randomization (scMR), virtual knockout screening, and AFADESI-MSI spatial metabolomics, we demonstrate that Sol transcends conventional single-target pharmacology. Instead, it operates as a pleiotropic modulator, orchestrating a highly compartmentalized restoration of lipid–inflammatory homeostasis, kinase signaling, and mitochondrial bioenergetics. Crucially, our multi-omics blueprint circumvents a major bottleneck in natural product research, namely target ambiguity, by directly mapping pharmacological execution to specific cell types and precise anatomical microregions.

A defining conceptual shift in contemporary neurodegeneration research is the recognition of PD not only as a proteinopathy but as a profound lipidopathy [1]. Our spatial metabolomics and co-enrichment analyses provide compelling in situ evidence that Sol directly targets this lipid–inflammatory axis. Specifically, the profound recovery of highly unsaturated phosphatidylcholines, such as PC(44:12), alongside cervonoyl ethanolamide (also known as synaptamide), highlights a highly specific membrane remodeling process. Cervonoyl ethanolamide is an endocannabinoid-like lipid mediator derived from docosahexaenoic acid (DHA, 22:6) that possesses potent anti-inflammatory and neurogenic properties [51]. The synchronized spatial depletion of these lipids following MPTP exposure, and their robust restoration in the striatum by Sol, signifies a targeted repair of membrane structural integrity and the re-establishment of anti-inflammatory lipid signaling. Furthermore, the suppression of phosphatidic acid (PA(37:2)), which is a lipid class often elevated during membrane degradation and cellular stress [52], indicates that Sol prevents pathological lipid catabolism rather than uniformly upregulating all lipid biosynthesis.

Beyond mapping spatial molecular fluctuations, a major advancement of our study lies in delineating the cellular “division of labor” driving this metabolic recovery. Traditional bulk omics inherently mask the cellular origin of therapeutic responses. By deploying scMR and in silico single-cell virtual knockout analyses (scTenifoldKnk), we unmasked a highly localized, cell-type-specific execution program for Sol. Within the neuronal compartment, candidate targets such as PRKCB and FABP5 were tightly linked to the restoration of mitochondrial translation and ATP synthesis-coupled electron transport. FABP5 is critical for the intracellular trafficking of hydrophobic ligands to mitochondria and nuclear PPARs, regulating bioenergetics and cell survival [53]. The virtual perturbation of these genes perfectly explains our spatial metabolomic observations: the in situ recovery of NADHX (a marker of NAD+ metabolic repair) and the reduction in elevated creatinine in the striatum, which collectively mirror a robust bioenergetic rescue. Given that Sol is a long-chain polyisoprenoid and a well-documented biochemical precursor for Coenzyme Q10 (a critical electron transport chain component) [54], its ability to rescue neuronal oxidative phosphorylation (OXPHOS) and mitochondrial redox pools represents a highly logical and potent mechanism against MPTP-induced Complex I inhibition.

However, our single-cell integration reveals that Sol’s efficacy clearly extends beyond neuronal confines, actively modulating the neuroglial and vascular microenvironments. The computational deletion of PRKCE and RORA in astrocytes and OPCs profoundly perturbed neuroinflammatory stress networks and trans-synaptic signaling. Astrocytic reactivity is a primary driver of neurotoxicity in PD [55], and RORA (Retinoic acid receptor-related orphan receptor alpha) is known to potently suppress glial NF-κB inflammatory pathways [56]. Simultaneously, PDGFRB modulation in vascular-associated cells disrupted blood–brain barrier adhesion and vascular morphogenesis. These findings suggest that Sol mitigates PD-like pathology through a synergistic multicellular network: rescuing neuronal bioenergetics while simultaneously disarming glial-driven neuroinflammation and repairing vascular niche integrity. Although microglia are an important type of immune/inflammatory cells in PD, the candidate signals related to microglia in this study were insufficient to support their use as the primary target for virtual knockout. Therefore, no extensive discussion was conducted on this matter.

Methodologically, the deployment of AFADESI-MSI provided indispensable orthogonal validation for these computational predictions. The brain is an exceptionally heterogeneous organ, and critical regional metabolic footprints are notoriously diluted in conventional tissue homogenates [57]. By retaining true cytoarchitecture, our spatial PLS-DA and ROI-based quantifications confirmed that Sol induces a distinct, region-selective metabolic reprogramming, as evidenced by the precise mapping of dopamine and L-DOPA recovery specifically in the striatum. The convergence of these in situ spatial footprints with single-cell transcriptomic predictions anchors our functional hypotheses in concrete, localized biochemical reality. Despite these comprehensive insights, several limitations warrant careful consideration. First, the MPTP mouse model is an acute toxicological paradigm that excels at recapitulating dopaminergic degeneration and motor deficits, but it does not fully capture the chronic progression or the endogenous α-synuclein aggregation characteristic of human PD. Second, an a priori sample-size and statistical power calculation was not performed; instead, the sample size (*n* = 3 animals per group) was determined based on well-established historical precedents in exploratory mass spectrometry imaging and spatial multi-omics literature to properly balance biological variation with analytical instrument throughput. To evaluate Sol’s efficacy against chronic synucleinopathy, future studies in transgenic models (such as A53T mutant mice) will be required. Additionally, although our single-cell virtual knockout analysis provides an advanced predictive framework for estimating downstream regulatory outcomes, it is nevertheless a computational perturbation. Future in vivo studies utilizing cell-type-specific Cre-loxP genetic targeting are essential to definitively confirm target engagement at the single-cell level. Finally, our spatially and cellularly resolved framework successfully prioritized several novel candidate targets, comprehensive full-panel validation at the transcript and protein levels was not performed for the entire spectrum of predicted genes. Consequently, conducting targeted qPCR and Western blot verification assays to systematically validate these computational predictions represents the immediate future direction of this research line [58].

In conclusion, our study not only elucidates the multifaceted, lipid–inflammatory mechanisms by which solanesol ameliorates PD-like pathology but also establishes a powerful new methodological paradigm. By bridging the gap between single-cell genetic prioritization, virtual perturbation, and spatial metabolic mapping, we provide a robust, cell-type-aware framework for decoding the complex neuropharmacology of natural products. It should be noted that existing modern therapeutics (e.g., Rasagiline, Pramipexole, Entacapone) are highly effective at symptomatically modulating synaptic dopamine levels; however, their clinical efficacy severely narrows or fails as dopaminergic neurons progressively go missing in late-stage disease, eliminating the vital presynaptic machinery. This profound clinical bottleneck underscores the urgent need for disease-modifying strategies. Solanesol, as an endogenous precursor for Coenzyme Q10 synthesis, offers a distinct disease-modifying therapeutic profile by directly targeting upstream mitochondrial dysfunction, bioenergetic failure, and compartmentalized spatial neuroinflammation. Its unique clinical promise lies in its potential to act as a multi-targeted neuroprotective agent that could be utilized in early-stage intervention or combination therapies alongside traditional dopamine-centric drugs to modify the disease trajectory.

## 5. Conclusions

In summary, this study establishes an integrated, spatially and cellularly resolved multi-omics framework that provides comprehensive insights into the neuroprotective mechanisms of Sol in MPTP-induced PD-like pathology. By seamlessly bridging brain single-cell genetic prioritization (scMR and transcriptomic localization) and in silico network perturbation (single-cell virtual knockout screening) with in situ AFADESI-MSI spatial metabolomics, we demonstrate that solanesol transcends conventional single-target paradigms to combat PD as a complex, systemic lipidopathy and bioenergetic disorder. Crucially, our findings unveil an elegant, cell-type-specific “division of labor” orchestrating therapeutic efficacy, whereby neuronal PRKCB and FABP5 dictate mitochondrial translational and respiratory recovery to directly counteract mitochondrial dysfunction, while astrocytic/OPC PRKCE and RORA disarm neuroinflammatory stress cascades, and vascular-associated PDGFRB preserves blood–brain barrier morphogenesis and matrix adhesion. This multicellular execution biochemically translates into a robust, compartmentalized restoration of the striatal microenvironment, manifested as the rebalance of dopaminergic (DA, L-DOPA) and cholinergic neurotransmission, the upregulation of protective structural/signaling lipids (cervonoyl ethanolamide and highly unsaturated PC species), and the repair of cellular redox networks (NADHX), coupled with the selective suppression of localized stress readouts (PA, creatinine, and thymidine). While the mass spectrometry platform utilized in this study captures total histochemical snapshots rather than active synaptic flux or real-time interstitial acetylcholine release, and the rodent MPTP model predominantly recapitulates rigidity and bradykinesia rather than the high-fidelity resting tremor characteristic of human Parkinson’s disease, the definitive causal link between in situ DA–ACh rebalancing and physical tremor alleviation is to be confirmed through future longitudinal electrophysiological studies. By anchoring single-cell gene regulatory dynamics directly to intact tissue microenvironments, this work not only clarifies the neuroprotective actions of solanesol but also offers a valuable analytical strategy for exploring the spatial and cellular mechanisms of natural neurotherapeutics.

## Data Availability

The original contributions presented in this study are included in the article/Supplementary Material. Further inquiries can be directed to the corresponding authors.

## Supplementary Materials

The following supporting information can be downloaded at: https://www.mdpi.com/article/10.3390/metabo16080541/s1, Table S1: Discriminating metabolites based on AFADESI-MSI data between the treatment groups and MPTP groups. Table S2: The values of R^2^X, R^2^Y, and Q^2^Y for PLS-DA model.
