# Supplementary material for: Spatial Metabolomics and Single-Cell Virtual Knockout Screening Reveal Solanesol Improves Parkinson’s Disease-like Pathology Based on Lipid Inflammation Mechanism
Source: Metabolites. 2026 Jul 31;16(8):541. doi: 10.3390/metabo16080541 (PMC13515215; doi:10.3390/metabo16080541)
Supplement: Supplementary file 1 [file metabolites-16-00541-s001.zip › metabolites-4447213-supplementary.pdf]

Table S1. Discriminating metabolites based on AFADESI-MSI data between the treatment groups and MPTP groups.

Optimal Name	Formula	Measured *m/z*	Theoretical *m/z*	Ion type	Product ions	ppm	P-value	FDR-corrected Q-value	Fold changes
Creatinine	$C_4H_7N_3O$	114.0660	114.0662	$[M+H]^+$	72.0474; 86.0741	1.75	3.22E-04	0.0021	1.42
Taurine	$C_2H_7NO_3S$	126.0220	126.0219	$[M+H]^+$	112.7720; 44.3720	0.79	8.90E-03	0.0115	0.63
Thymidine	$C_{10}H_{14}N_2O_5$	243.0990	243.0983	$[M+H]^+$	127.0500; 117.0546; 84.0444; 110.0237	3.1	3.64E-03	0.0058	1.63
5-Methylcytidine	$C_{10}H_{15}N_3O_5$	258.1091	258.1088	$[M+H]^+$	126.0662; 109.0397; 133.0495; 83.0604	1.37	4.46E-04	0.0021	0.68
Cervonoyl ethanolamide	$C_{24}H_{36}O_3$	395.2552	395.2535	$[M+Na]^+$	62.0600; 311.2365; 354.2797	4.47	2.40E-04	0.0021	0.73
NADHX	$C_{21}H_{31}N_7O_{15}P_2$	706.1310	706.1278	$[M+Na]^+$	143.0821; 346.0553	4.6	2.58E-03	0.005	0.49
PC(O-31:0)	$C_{39}H_{80}NO_7P$	728.5564	728.5538	$[M+Na]^+$	184.0733; 669.4836; 545.4915; 241.2168; 466.3298	3.65	2.75E-03	0.005	0.71
PA(37:2)	$C_{40}H_{75}O_8P$	732.5537	732.5509	$[M+NH4]^+$	335.2945; 241.2186; 377.2079	3.93	9.04E-05	0.0014	2.33
PC(33:3)	$C_{41}H_{76}NO_8P$	764.5200	764.518	$[M+Na]^+$	184.0733; 683.4652; 541.4621	2.71	1.19E-03	0.0033	0.35
PC(36:5)	$C_{44}H_{78}NO_8P$	780.5540	780.5539	$[M+H]^+$	184.0742; 721.4811; 597.4885	0.15	2.23E-03	0.005	0.54
PC(44:12)	$C_{52}H_{80}NO_8P$	916.5225	916.5193	$[M+K]^+$	184.0732; 819.4955; 695.5038	3.56	1.02E-03	0.0030	0.52c
PC(38:4)	$C_{46}H_{84}NO_8P$	848.5566	848.556	$[M+K]^+$	184.0733; 751.5383; 627.5355	0.72	1.36E-03	0.0042	0.83
PC(P-38:5)	$C_{46}H_{82}NO_7P$	792.5928	792.5901	$[M+H]^+$	184.0739; 733.5162; 609.5254; 303.2324; 492.3454	3.41	4.78E-02	0.0478	1.75

Table S2. The values of R^2X, R^2Y, and Q^2Y for PLS-DA model.

Region_model	R^2X	R^2Y	Q^2Y
ALL_neg	0.45	0.88	0.62
ALL_pos	0.43	0.89	0.58
STR_neg	0.42	0.87	0.66
STR_pos	0.47	0.83	0.61
